# Aptasensors for rapid detection of *Escherichia coli* O157:H7 and *Salmonella typhimurium*

**DOI:** 10.1186/1556-276X-7-658

**Published:** 2012-11-29

**Authors:** Wen-he Wu, Min Li, Yue Wang, Hou-xian Ouyang, Lin Wang, Ci-xiu Li, Yu-chen Cao, Qing-he Meng, Jian-xin Lu

**Affiliations:** 1 Key Laboratory of Laboratory Medicine, Ministry of Education, Zhejiang Provincial Key Laboratory of Medical Genetics, Wenzhou Medical College, Wenzhou, Zhejiang, 325035, China; 2 Department of Laboratory Medicine, The University of Texas, MD Anderson Cancer Center, Houston, Texas, 77030, USA

**Keywords:** Colorimetric detection, Aptasensor, *E*. *coli* O157:H7, *Salmonella typhimurium*, AuNPs

## Abstract

Herein we reported the development of aptamer-based biosensors (aptasensors) based on label-free aptamers and gold nanoparticles (AuNPs) for detection of *Escherichia coli* (*E*. *coli*) O157:H7 and *Salmonella typhimurium*. Target bacteria binding aptamers are adsorbed on the surface of unmodified AuNPs to capture target bacteria, and the detection was accomplished by target bacteria-induced aggregation of the aptasensor which is associated as red-to-purple color change upon high-salt conditions. By employing anti-*E*. *coli* O157:H7 aptamer and anti-*S. typhimurium* aptamer, we developed a convenient and rapid approach that could selectively detect bacteria without specialized instrumentation and pretreatment steps such as cell lysis. The aptasensor could detect as low as 10^5^colony-forming units (CFU)/ml target bacteria within 20 min or less and its specificity was 100%. This novel method has a great potential application in rapid detection of bacteria in the near future.

## Background

Diarrheal diseases caused by a range of enteropathogenic bacteria represent a major health threat in developing countries. According to the data from the World Health Organization, there are almost two million deaths per year (1.7-2.5 million deaths) caused by diarrhea. Emerging and known enteropathogenic bacteria are, among others, *Escherichia coli* (*E*. *coli*) O157:H7 and *Salmonella typhimurium* (*S. typhimurium*). *E*. *coli* O157:H7 has been recognized as a major intestinal flora for a long time since it was detected as the pathogen that causes foodborne disease outbreaks in the USA [1,2]. In the past decades, *E*. *coli* O157:H7 has received tremendous attention, because it becomes an important pathogenic cause for several severe illnesses in human beings such as gastrointestinal disease and bloody diarrhea which is a root cause of hemolytic uremic syndrome [3]. In addition, the incidence of *S. typhimurium* outbreaks is on the rise. In 2010, the outbreak of *S. typhimurium* resulted in 85 infections according to the data from the Centers for Disease Control and Prevention (CDC, USA). Most recently, a multi-state outbreak in the USA was associated with contaminated ground beef.

With the health risks of enteropathogenic bacteria, various methods have been developed for their analysis. Traditional culture-based methods for assay of *E*. *coli* O157:H7 and *S. typhimurium*, however, are time-consuming and are unable to meet the needs of real-time bacteria detection. Other detection technologies such as immunoassays [4,5] and polymerase chain reaction-based assay [6] require either long time spans or specialized instrumentation. Therefore, it is critical to develop a fast and simple method to detect enteropathogenic bacteria for the diagnosis and treatment.

Aptamer is single-stranded nucleic acid (DNA or RNA) ligand that usually possesses high affinity and results in a significant conformation change upon binding with a wide range of targets. Aptamers are generally selected from the pools containing randomly created sequences through an *in vitro* systematic evolution of ligands by exponential enrichment (SELEX) [7]. Compared to antibody-based biosensors, aptamer-based biosensors (aptasensor) [8,9] possess unprecedented advantages with high productivity, affinity, selectivity, and stability.

Recently, several aptasensor using gold nanoparticles (AuNPs) [10,11] that act as signal transducer element of the biosensor [12] have been developed. The application of single-stranded DNA-modified AuNPs for the highly selective colorimetric detection has been conducted, in which it can result in an aggregation of AuNPs with red to pinkish/purple color change in the presence of target molecules in solution [13]. Wei et al. reported a simple and sensitive aptamer-based colorimetric sensor of thrombin using unmodified AuNPs [14]. The thrombin aptamer (TBA) was used to form aptamer-AuNPs. Introduction of thrombin leads to the conformation change of aptamer and increases the repulsion between TBA and AuNPs causing salt-induced aggregation.

Nevertheless, most of AuNPs-based aptasensors were developed to detect proteins and small molecules [15,16]. To the best of our knowledge, no work exists in AuNPs-based aptasensors for bacteria detection. Herein, we reported the development of aptamer-based biosensors (aptasensors) based on label-free aptamers and AuNPs for the detection of *E*. *coli* O157:H7 and *S. typhimurium*, with the aim of establishing a preliminary method to evaluate the utility of aptamer-AuNPs assay for enteropathogenic bacteria. Two species of bacteria (*E*. *coli* O157:H7 and *S. typhimurium*) were examined in this work.

## Methods

### Reagents and chemicals

All DNA oligonucleotides were synthesized and HPLC purified by Bioneer Co. Ltd. (Daejeon, South Korea). The sequences of these oligonucleotides are shown in Table 1. We truncated the sequences of anti-*E*. *coli* O157:H7 aptamer and anti-*S. typhimurium* aptamer by prediction of the secondary structure using web-based Vienna RNA software. AuNPs (approximately 15 nm diameter) were purchased from Fitzgerald Industries International (MA, USA). Sodium chloride (NaCl), potassium chloride (KCl), disodium hydrogen phosphate (Na_2_HPO_4_), and potassium dihydrogen phosphate (KH_2_PO_4_) were purchased from China National Pharmaceutical Group Corporation (Shanghai, China). All chemicals were of analytical grade. The water used throughout the experiments was purified by a Milli-Q system (Millipore, Bedford, MA, USA).

**Table 1:** Sequences of oligonucleotides employed in this work

**Name**	**Sequence**
A1: anti-*E*. *coli* O157:H7 aptamer	5^′^-CCGGACGCTTATGCCTTGCCATCTACAGAGCAGG
	TGTGACGG-3^′^
A2: anti-*S. typhimurium* aptamer	5^′^-CCAAAGGCTACGCGTTAACGTGGTGTTGG −3^′^
R1: random sequence	5^′^-ATCCATGGGGCGGAGATGAGGGGGAGGAGGGCG
	GGTACCCGGTTGAT-3^′^

The sequences of specific target bacteria binding aptamers [17] were truncated after prediction of the secondary structure using web-based Vienna RNA software.

### Bacterial strains and cultivation

*E*. *coli* O157:H7 (CICC21530) was purchased from China Center of Industrial Culture Collection (CICC, Beijing, China). *E*. *coli* O111 (CMCC44151) was purchased from National Center for Medical Culture Collections (CMCC, Beijing, China). *S. typhimurium* (CMCC50115), *Shigella flexneri* (CMCC51571), *Salmonella paratyphi* A (CMCC50001), *S. paratyphi* B (CMCC50004), *E*. *coli* (ATCC25922), *E*. *coli* (CMCC44825), *Pseudomonas aeruginosa* (ATCC27853), *Listeria monocytogenes* (ATCC19115), and *Staphylococcus aureus* (ATCC25923) were gifts from the Central Laboratory of Biology of Wenzhou Medical College (Zhejiang, China). Stock cultures in 25% glycerol were maintained frozen at −80°C.

All bacteria were inoculated into lysogeny broth (LB) and grown for 6 h at 37°C with shaking at 165 rpm. The cultures containing bacteria were centrifuged at 3,000 rpm for 5 min and washed with phosphate-buffered solution (PBS) (10 mM, pH 7.4) three times. The pellets were then dispersed in PBS. Serial dilutions of cultures were made in PBS, 50 μl diluted suspension was inoculated onto agar plates for enumeration. The bacterial densities were determined using a scattered light turbidimeter. The actual amount of bacteria was then determined based on the bacterial density.

### Instrumentation

Ultraviolet spectroscopy was performed on aqueous solutions of DNA aptamer using Nanodrop 2000 Spectrophotometer (Thermo Fisher Scientific, USA) at 260 nm. Colorimetric assays were recorded on Varioskan Flash spectral scan multimode plate reader (Thermo Fisher Scientific, USA). Scanning electron microscopy (SEM) images were taken on the ZEISS-ULTRA 55 (ZEISS, Oberkochen, Germany) with an accelerating voltage of 5.0 kV. The pH measurements were carried out on model PHS-3E digital ion analyzer (Jiangsu Instruments, China). Zeta potential measurements were performed using the NICOMP 380ZLS zeta potential/particle sizer (Agilent Technologies, CA, USA).

### Preparation and characterization of aptasensors

Aptamers play an important role in colorimetric detection. Since aptamers can absorb AuNPs and stabilize AuNPs against aggregation upon addition of high level salts, it is essential to determine an optimal amount of aptamers. In our study, aptamer-AuNPs were prepared according to previous work [18]. Briefly, aptamers (A1 or A2) of different concentrations were mixed with 50 μl AuNPs thoroughly and allowed to react at room temperature for 25 to 30 min. The above solution was incubated at room temperature for 10 min following addition of 50 μl PBS. Subsequently, 5 μl of NaCl (1.7 M) was mixed with the resulting solution. After equilibrating for 10 min, the UV–vis absorption spectrum was measured at the wavelength range from 450 to 750 nm with Varioskan Flash spectral scan multimode plate reader. All assay procedures were performed at room temperature. The quality of aptamer-AuNPs was monitored with SEM and zeta potential analyzer.

### Colorimetric detection of *E. coli* O157:H7 and *S. typhimurium*

Various amounts of target bacteria of 50 μl were added to the 50 μl aptamer-AuNPs solution. After incubating for 10 min, 5 μl of NaCl (1.7 M) was introduced to the mixtures, followed by either visual observation or UV–vis characterization. A series of bacterial exposure to the aptamer-AuNPs were carried out to explore the specificity of the aptamers. The bacteria were as follows: *E*. *coli* (ATCC 25922, CMCC44825, and CMCC44151), *S. paratyphi* A, *S. paratyphi* B, *S. flexneri*, *P. aeruginosa*, *L. monocytogenes*, *S. aureus*, *S. typhimurium*, and *E*. *coli* O157:H7.

### Statistical analysis

Results were expressed as mean ± SD. Comparisons between two groups were made by unpaired two tailed Student’s *t* test using SPSS 15.0 software. The *P* value of less than 0.05 was taken to indicate statistical significance.

## Results and discussion

**Figure 1:**
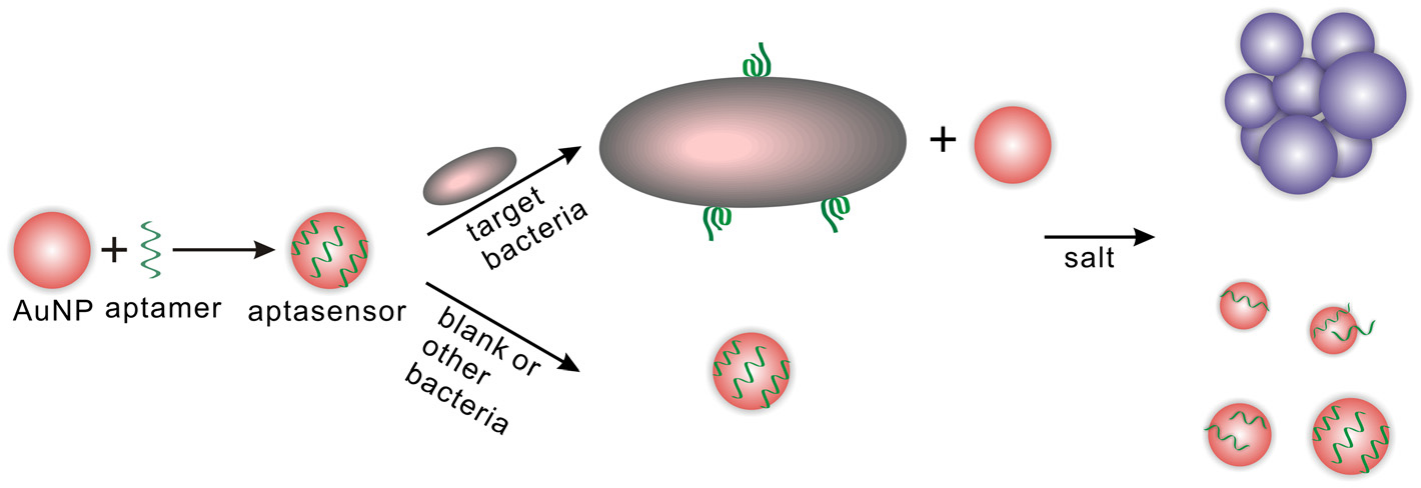
Sketch of the experimental principle of target bacteria detection using aptamer and AuNP.

We used aptamers as specific recognition elements and unmodified AuNPs as signal transducer element. A schematic description is shown in Figure 1. The 15-nm AuNPs are red-colored due to its surface plasmon resonance absorption located at 520 nm [19]. Target bacteria binding aptamers were adsorbed to AuNPs’ surface and prevented AuNPs from salt-induced aggregation via electrostatic repulsion. However, the conformation of aptamers changed upon the addition of target bacteria, which made aptamers separate from AuNPs. Due to the reduced ability of protecting AuNPs against salt-induced aggregation and the interparticle-coupled plasmon excitons [20,21], the color change of the AuNPs from red to purple can be observed after addition of salt.

### Optimal condition screening of aptamer for binding with AuNPs

**Figure 2:**
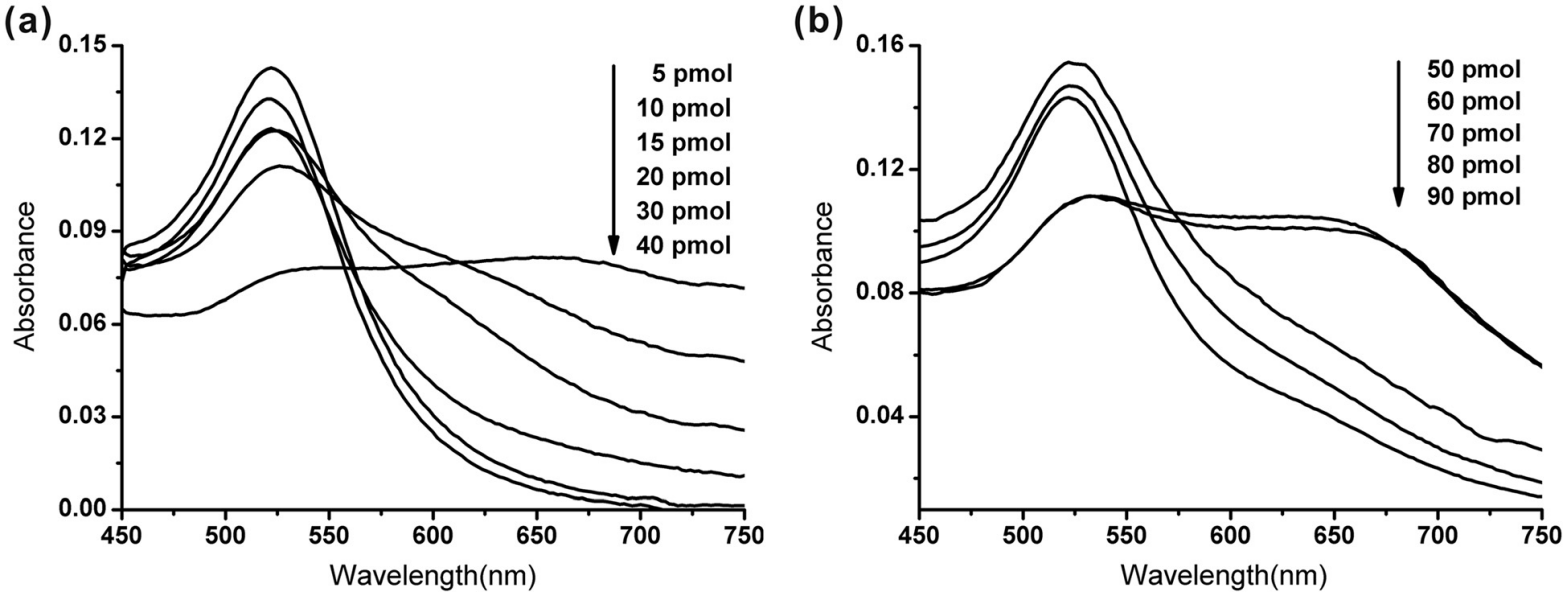
Optimization of (a) A1 amounts and (b) A2 amounts.

To establish an optimal ratio of aptamer to AuNPs at which the aptamer-AuNPs remain stable in the absence of target bacteria after addition of high level salt, different amounts of aptamer were examined. A1 with different amounts at 5, 10, 20, 30, and 40 pmol and A2 with different amounts at 50, 60, 70, 80, and 90 pmol were used. As shown in Figure 2a, there was only one peak located at 520 nm for 30 pmol A1 or more, while the 520-nm peak shifted to 525 nm and a new, broad absorption (600 to 750 nm) appeared when the amount of A1 was less than 20 pmol. It indicated that little aggregation of AuNPs-DNA occurred upon an addition of 30 pmol A1 or more, whereas significant aggregation of AuNPs was observed when the amounts of A1 were below 20 pmol. We also experimentally estimated that the optimal amount of A2 was 80 pmol (Figure 2b). To stabilize the AuNPs solution against aggregation, hydrophobic interaction between the bases and the AuNP is essential to cause aptamers to stick to the AuNPs [22]. For this reason, the length of A1 (33 mer) and A2 (29 mer) should be responsible for the discrepancy observed. Therefore, the 30 pmol A1 and 80 pmol A2 were chosen in the subsequent work.

### Characterization of aptasensors

**Figure 3:**
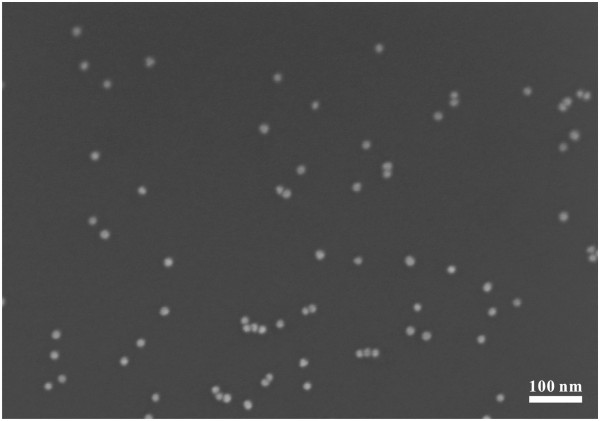
**SEM images of A2-AuNPs.** Scale bar is 100 nm.

The success of the assembly was confirmed by SEM and zeta potential measurements. As shown in Figure 3, after assembly of A2-AuNPs, we observed that all the aptasensors were spherical in shape with an average size of 15 nm, with no change in the morphology and were well-separated. The A2-AuNPs kept the original red color and could be used in subsequent detection. The zeta potential of AuNPs was −30.7 ± 3.7 mV (*n* = 3) owing to the presence of citrate ions on the surface of AuNPs. After interaction, zeta potential of A2-AuNPs substantially increased to −21.8 ± 3.1 mV (*n* = 3). The increase in surface-exposed negative charges indicates the formation of the aptamer-AuNPs complex.

### Colorimetric detection of *E. coli* O157:H7 with aptamer-AuNPs

**Figure 4:**
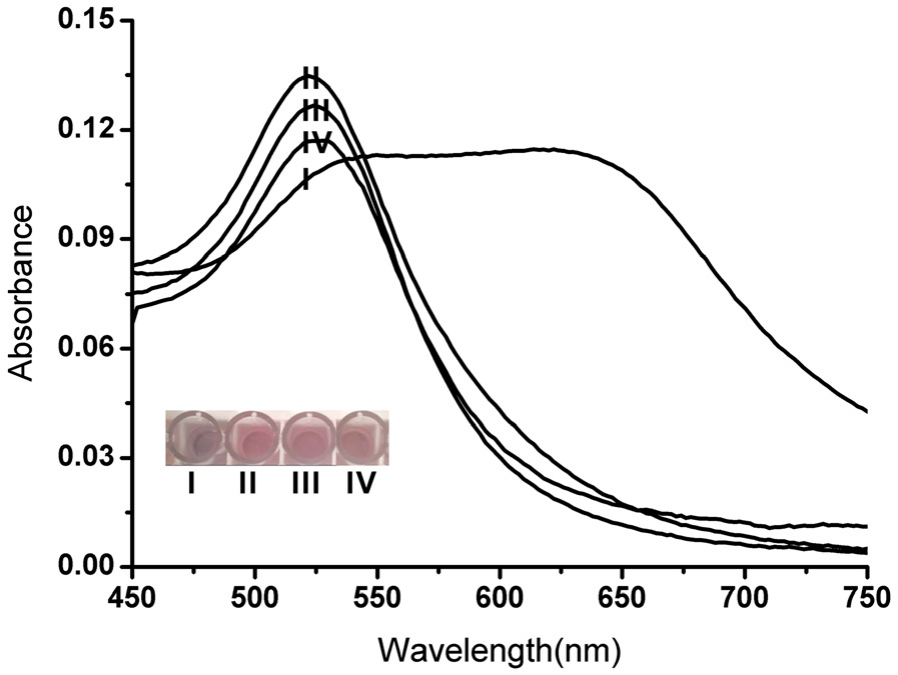
**UV–vis absorption spectra and colorimetric response.** (I) *E*. *coli* O157:H7 with A1/AuNPs. (II) PBS with A1/AuNPs. (III) *E*. *coli* O111 with A1/AuNPs. (IV) *E*. *coli* O157:H7 with R1/AuNPs.

A positive control was investigated with *E*. *coli* O157:H7 (10^8^ CFU/ml) and A1-AuNPs. The PBS buffer was used instead of *E*. *coli* O157:H7 as a reagent blank. *E*. *coli* O111 (10^8^ CFU/ml) with A1/AuNPs and *E*. *coli* O157:H7 (10^8^ CFU/ml) with R1/AuNPs served as the negative controls. As shown in Figure 4, in the presence of *E*. *coli* O157:H7, A1 bind to *E*. *coli* O157:H7 and form a structured complex, resulting in the aggregation of AuNPs upon salt addition that leads to the characteristic red-to-purple color change (Figure 4, I). In contrast, AuNPs remain stable and red in color because of the unbound A1 (Figure 4, II and III) or R1 (Figure 4, IV) on the AuNPs. UV–vis absorption spectra also correlated well with these visual observations. Significant change in absorption was only observed for *E*. *coli* O157:H7 with A1-AuNPs.

### Sensitivity analysis

**Figure 5:**
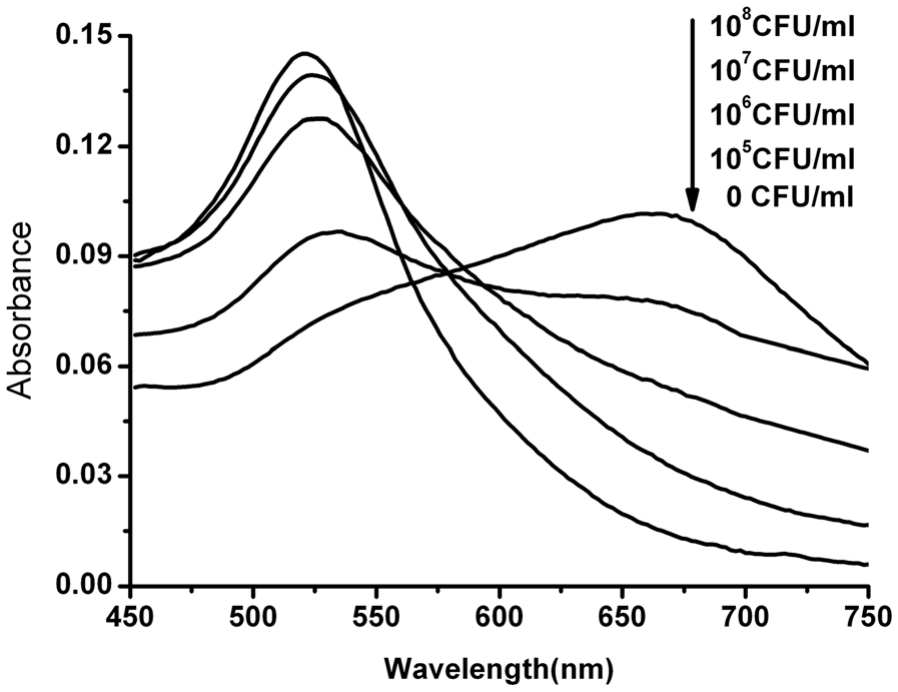
**UV–vis adsorption spectra for aptasensor-based detection before and after the addition of *E*. *coli *O157:H7.** From top to bottom are 10^8^, 10^7^, 10^6^, 10^5^, and 0 CFU/ml *E*. *coli* O157:H7.

Sensitivity analysis was determined by monitoring the absorbance change following the addition of various amounts of bacteria and high level of salt. As shown in Figure 5, along with the increase of the *E*. *coli* O157:H7 concentration, the absorbance at 520 nm gradually shifted and decreased, while the broad absorption (600–750 nm) gradually increased.

**Figure 6:**
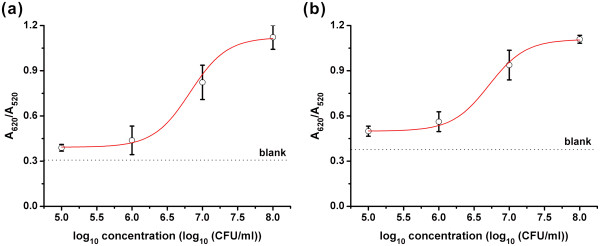
**Plot of bacterial concentration vs. their absorbance ratio.** (**a**) Plot of *E*. *coli* O157:H7 concentration vs. absorbance ratio (A_620_/A_520_) for the *E*. *coli* O157:H7 assay. (**b**) Plot of *S. typhimurium* concentration vs. absorbance ratio (A_620_/A_520_) for the *S. typhimurium* assay. Error bars represent standard deviations from three independent assays.

A series of reactions containing various amounts of target bacteria were employed to produce a calibration curve for the *E*. *coli* O157:H7 or *S. typhimurium* assay (Figure 6). In our experiments, the absorbance peak ratio at 620 and 520 nm was employed to quantify the color change of the aptamer-AuNPs. Though a relatively low signal, the absorption ratio (A_620_/A_520_) of 10^5^ CFU/ml *E*. *coli* O157:H7 or *S. typhimurium* was still significantly higher than that of blank (three times the standard deviation of blank), suggesting sufficient sensitivity of this assay to detect *E*. *coli* O157:H7 or *S. typhimurium*.

### Specificity analysis

**Figure 7:**
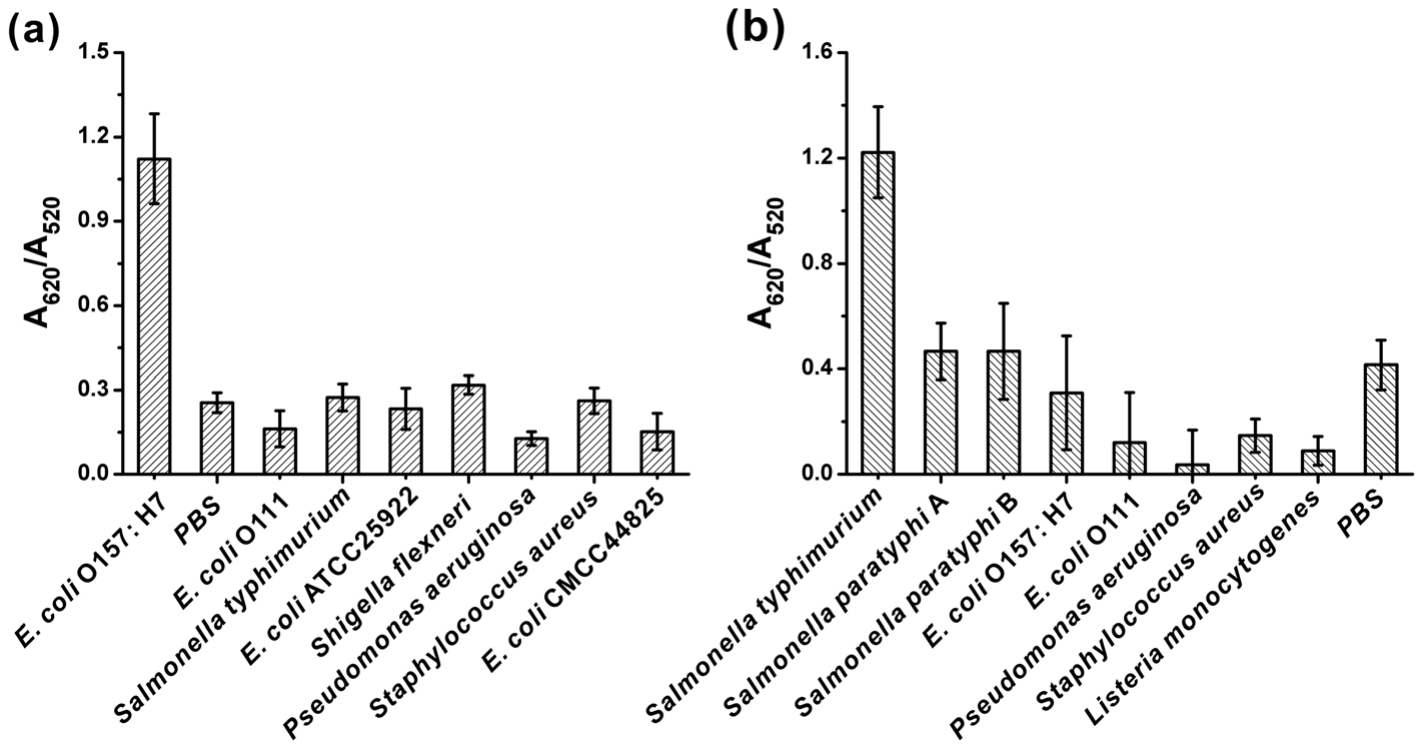
**Specificity of aptasensor-based detection.** (**a**) Specificity of A1/AuNPs detection in *E*. *coli* O157:H7. (**b**) Specificity of A2/AuNPs detection in *S. typhimurium.*

To ensure that such a color change was only specific to the binding of target bacteria with the aptamer, the specificity of the aptasensor was investigated on a series of other bacteria such as *S. flexneri*, *S. paratyphi* A, and *S. paratyphi* B. As shown in Figure 7a, the A_620_/A_520_ ratio of A1/AuNPs in the presence of *E*. *coli* O157:H7 was four times more than of those for other bacteria. Similarly, these changes were minor with all the other bacteria except *S. typhimurium* for A2/AuNPs (Figure 7b). The A_620_/A_520_ difference between these bacteria demonstrated that this aptasensor could determine target bacteria with high specificity, while other bacteria could not separate aptamers from AuNPs effectively and exhibited little change in color.

## Conclusions

By using both anti-*E*. *coli* O157:H7 aptamer and anti-*S. typhimurium* aptamer as models, we have developed aptasensors-based, unmodified AuNPs that can rapidly and selectively detect bacteria with the naked eye. The aptasensors take only 20 min or less to complete bacteria detection, which greatly reduces the time compared with conventional bacterial cultivation and other rapid detection methods [23,24]. In addition, The detection limit (10^5^ CFU/ml) was significantly lower [25,26] than or comparable [23] to the presently available antibody-based biosensors with the same bacteria. In order to meet lower levels of bacteria detectable, sample preconcentration steps will be necessary prior to analysis, such as microfiltration and immunomagnetic separation. In addition, this method avoids the labeling of aptamers or modification of AuNPs, which significantly reduces the cost. This novel method is simple and can be used for a rapid diagnosis for diarrheal diseases. However, the most important strength of this aptasensor is its direct detection of the whole bacteria without specialized instrumentation and pretreatment steps such as cell lysis. Moreover, this method could be potentially applied in principle to detect other bacteria by substituting the anti-*E*. *coli* O157:H7 aptamer or anti- *S. typhimurium* aptamer.

## Competing interests

The authors declare that they have no competing interests.

## Authors’ contributions

WHW and JXL conceived and designed the experiments. ML, YW, HXO, LW, CXL, and YCC performed the experiments. ML, YW, and HXO analyzed data. JXL and QHM contributed materials and analysis tools. WHW, JXL, and QHM wrote the manuscript. All authors read and approved the final manuscript.
